# Dispensing Practices for Weight Management Products in Eastern Saudi Arabia: A Survey of Community Pharmacists

**DOI:** 10.3390/ijerph182413146

**Published:** 2021-12-13

**Authors:** Mahmoud E. Elrggal, Sarah Ibrahim Alamer, Saad A. Alkahtani, Mohammed Ahmed Alshrahili, Adnan Alharbi, Bayan Ali Alghamdi, Mohammad Fathullah Zaitoun

**Affiliations:** 1 Clinical Pharmacy Department, College of Pharmacy, Umm Al-Qura University, Makkah 21955, Saudi Arabia; elrggalme@gmail.com (M.E.E.); assharbi@uqu.edu.sa (A.A.); 2 Pharmacy Practice Department, College of Clinical Pharmacy, King Faisal University, Al Hofuf 31982, Saudi Arabia; Sialamer@kfu.edu.sa; 3 Clinical Pharmacy Department, College of Pharmacy, Najran University, Najran 55461, Saudi Arabia; Saad.rkh@gmail.com; 4 Pharmacy Department, Armed Forces Hospital Jazan, Jazan 84224, Saudi Arabia; alshrahili2020@icloud.com; 5 Pharmaceutical Care Administration, Armed Forces Hospitals Southern Region, Khamis Mushait 61961, Saudi Arabia; bayanali4@hotmail.com

**Keywords:** obesity, weight reduction, weight loss, anti-obesity agents, supplements, dietary, over-the-counter drugs, community pharmacy

## Abstract

Due to changing lifestyles and socioeconomic status, obesity prevalence has been rising in Saudi Arabia, and community pharmacists often counsel patients about its management. The study aimed to evaluate practices of community pharmacists involved in dispensing products for weight control in four cities located in the eastern province of Saudi Arabia. A cross-sectional study was conducted involving community pharmacists in Dammam, Dhahran, Khobar, and Al-Ahsa, using a Likert format questionnaire. Only those who consented to participate were handed the questionnaire. A total of 100 complete responses were analyzed. The median value for packs sold per month for tea containing products Al-Diafa Slimming Tea, Jamue Tea, and Green Tea was ≥6 while the same for orlistat and apple cider vinegar were ≤4. Moreover, >50% of pharmacists mentioned that orlistat and apple cider vinegar were effective while ≥35% mentioned that metformin and Jamue tea were effective. Furthermore, ≥25% mentioned that green tea and Al-Diafa slimming tea were effective. Excluding orlistat, >50% of pharmacists did not know about adverse effects for other products. The rate of dispensing of several weight loss products was significant for participants’ background characteristics, such as time duration of consultation, gender, and age of patients, and pharmacist work experience (*p* < 0.05). The tea products and orlistat were the most frequently sold products, and community pharmacists appeared most knowledgeable about the effectiveness and adverse effect of orlistat. The pharmacists seemed to be aware about the effectiveness of other weight loss products; however, their knowledge about their potential adverse effects was unsatisfactory.

## 1. Introduction

Obesity is a preventable medical condition characterized by an excessive accumulation of body fat; it is now considered a disease in itself [1,2,3]. Obesity could negatively impact an individual’s health [4]. It is regarded as one of the most pressing epidemic conditions and is assumed to threaten public health [3]. Obesity is associated with multiple morbidities and chronic illnesses, such as disabilities, oncologic, cardiovascular, and endocrine diseases, and mental illnesses, such as depression. Such morbid conditions further increase the chances of mortality [2,3].

The prevalence of obesity has been on the rise, especially in developing countries, such as Saudi Arabia, owing to changing lifestyles and socioeconomic status. According to recent literature, a prevalence of 24.7% for a body mass index (BMI) > 30 kg/m^2^ was reported [5]. The improved Saudi socioeconomic status was associated with a sedentary lifestyle and increased consumption of processed foods, sugar-sweetened beverages, etc. These have led to a surge in obesity cases [6,7,8,9]. 

Dietary supplements are among the most common remedies used for obesity. These products contain natural products or derivatives that provide nutritional support or prevent adverse health outcomes [3,10]. In addition, anecdotal evidence may be available concerning the clinical effectiveness and long-term safety of such products [3,10]. Besides, they are regulated differently than most medication products [11]. This may be the case with supplements used for weight loss.

Pharmacists are considered experts when it comes to knowledge about medications. Community pharmacists are accessible to the public and are usually regarded as the first and, at the same time, last point of contact between a patient and the healthcare system [12,13]. Moreover, pharmacists acquire considerable patient education and counseling skills that cover most medical conditions during their training. Such conditions do include education about obesity and its management [14]. Hence, pharmacists are usually considered empowered in counseling patients about obesity management [13,14]. 

Several weight control products are available in Saudi Arabia, such as orlistat, a prescription-only-medication (POM) [15]. While others, such as teas, herbs, dietary supplements, etc., are available as over-the-counter (OTC). This study aimed to evaluate current practices of community pharmacists involved in dispensing/selling weight control products in four cities in eastern Saudi Arabia.

## 2. Methods

A cross-sectional study was conducted for three months, targeting pharmacists working in community pharmacies in the country’s eastern region. The study involved the cities of Dammam, Dhahran, Khobar, and Al-Ahsa. Licensed community pharmacists with at least six months of work experience were eligible to participate in the study. The sampling method selected for the study was convenience sampling, as it provided the means to collect a larger number of responses from pharmacies. It was done by contacting the pharmacies located in vicinities nearby the researchers. A sample of community pharmacists working across four cities was targeted. According to a previous study, there were roughly 75 pharmacists per 100,000 population in the eastern region [16].

A questionnaire was developed for this study by receiving feedback from 25 community pharmacists working at different study sites. First, the pharmacists were asked to review the questionnaire drafts in terms of its relevance to the topic, content, ease of understanding, suggestions for further improvement, etc. This helped in the identification of the supplements commonly used for weight loss. A structured questionnaire was subsequently prepared based on the feedback provided by those panelists. The purpose of the questionnaire was to capture the knowledge and practice of community pharmacists involving such products. A secondary objective was to identify the most commonly sold products at those study sites. The language in which the questionnaire was developed was English. The format selected for the items was a Likert format having options from 1 (effective) up to 4 (not effective) to rate the effectiveness of such products. In addition, some open-ended items were included to identify the most frequently sold products and anticipated adverse effects of those products. 

The questionnaire was divided into several sections, namely demographics, including gender, work experience, and work location. The second section focused on documenting the pharmacist’s knowledge and perception regarding the safety and effectiveness of the weight loss supplements. Finally, the third section focused on the most frequently sold weight loss products. Hence, the objectives were to measure the average selling rate of weight loss products, reported side effects, and pharmacists regarding the effectiveness of the supplements in promoting weight loss. 

The pharmacists working in the community pharmacies were approached during regular working hours on weekdays and were briefed about the study. The voluntary nature of the study was explained. Before the data collection, the participants consented. Only those who agreed to participate were handed the questionnaire. The questionnaire underwent face validation, and its reliability was assessed using Cronbach’s alpha. It is worth noting that orlistat and metformin were prescription-only medications (POM) while others were over-the-counter (OTC) products. Therefore, the terms ‘dispensing’ and ‘selling’ are used throughout the manuscript.

The data collected were entered in Microsoft Excel and later imported into Statistical Package for Social Sciences version 23 for analysis. Descriptive statistics, such as mean and standard deviation, were used for continuous data, while frequency and percentage were used for categorical data. Based on the outcomes selected for the analysis, the variables of time of consultation, age of patients, gender of patients, pharmacist’s work experience were identified as independent variables (IV). In contrast, the rate of weight loss products selling per month was considered a dependent variable (DV). The Mann–Whitney U test was used to report significance in OTC product selling rates, based on independent variables of the participants’ characteristics. The study was approved by an ethics committee affiliated with the Saudi Ministry of Health.

## 3. Results

A total of 101 pharmacists responded to the survey. One response was incomplete and therefore excluded, leaving 100 complete responses for analysis. The Cronbach’s alpha value was 0.513 (N = 38 items) for the questionnaire. The summary of background characteristics is presented in Table 1.

The average packs sold in a month for several supplements used for weight loss is presented in Table 2.

The pharmacists were asked about the effectiveness of these products. Most pharmacists mentioned orlistat (N = 71, 71%) and apple cider vinegar (N = 50, 50%) to be always effective. The results are presented in Table 3.

Regarding adverse effects of these products, most notable was the reporting of GIT adverse effects of orlistat (N = 43, 43%) followed by GIT adverse effects (N = 21, 21%) and hypoglycemia (N = 16, 16%) for metformin. Except for orlistat, >50% of pharmacists did not know about adverse effects for other products in question. The results are summarized in Table 4.

The dependent variable (DV) of the ‘rate of monthly dispensing/selling’ of products, FormoLine, orlistat, chromium, Opuntia capsules, diet power, apple cider vinegar, Jamue tea, Al-Diafa tea, and green tea, was significant (*p* < 0.05) for the independent variable (IV) of ‘time during of consultation’. The dispensing/selling rate for all significant products mentioned above was higher when the consultation duration was more than 5 min. However, the dispensing/selling rate for products’ metformin’ and ‘ripped power’ was higher when the consultation duration was less than or up to 5 min. 

## 4. Discussion

This study was conducted to document the practices of community pharmacists about the supplements used for weight loss. The study was conducted in community pharmacies located in Khobar, Dammam, Dhahran, and Al-Ahsa. All are cities located in the eastern region of Saudi Arabia. Previously, a study conducted in this region reported the sale of supplements that lacked evidence of efficacy and safety. Therefore, the study called for further educational engagement and awareness creation [17]. In addition, another study conducted in the Aseer region observed that though pharmacists working in the community pharmacies had a positive attitude towards such products, there was a knowledge deficit among pharmacists regarding FDA approval for such products [3]. Hence, it was clear that the community pharmacists who participated in those studies lacked sufficient knowledge in this domain regardless of their work region.

It was observed in the study that almost all participants were male. It is pertinent to mention that male pharmacists dominate the Saudi community pharmacy landscape; a previous study by Alshahrani S.M. reported 98% male pharmacists. Hence, our findings are in line with Alshahrani’s findings [3]. Furthermore, it was observed in the study that there was almost an equal sample of pharmacists with up to 5 years of work experience and above five years. However, in Alshahrani’s study, most pharmacists had up to 3 years of experience [3].

It was observed that the most frequently sold products were a variety of teas. According to the report of Market Research, the retail sale value of the tea in the Saudi market amounted to more than USD 850 million. Despite the pandemic, the tea market retail sale value in Saudi Arabia is predicted to attain a level above USD 1 billion in the next five years [18]. In addition, Alshahrani also reported green tea as the most commonly used weight loss product [3]. One of the reasons for this occurrence could be the tendency to use natural herbs instead of medications for weight loss. Moreover, increased use of tea may also result from cultural norms. However, the reason behind the use of tea as a weight loss agent among Saudis needs to be investigated.

It was observed that the average sale of orlistat was four boxes per month. Alshahrani also reported a roughly 6–7% proportion of the patients prescribed the same [3]. Most pharmacists mentioned that orlistat was an effective obesity medication. In this regard, a study has shown a significant weight reduction mediated by orlistat and recommended its use with other measures, including lifestyle modifications [19]. In addition, community pharmacists’ most commonly reported orlistat adverse effects were related to the gastrointestinal tract, such as diarrhea, bloating, and fatty/oily spots in undergarments. This finding was similar to the previously reported adverse effects [20]. In addition, the most common adverse effect reported by the participants for metformin was hypoglycemia. In this regard, it is important to mention that maintenance of blood glucose is a desired effect of metformin [21].

Other products were either not available or not sold in numbers that could provide meaning to our findings. Reflecting on the responses of community pharmacists regarding the adverse effects of such products, it was evident that pharmacists were at most times correct in identifying adverse effects of commonly sold products, such as orlistat. Further studies are recommended to compare the knowledge competence of pharmacists in weight loss products by comparing subjective knowledge with the available evidence. Hence, it could be said that pharmacists tend to keep abreast with knowledge regarding commonly sold products. According to IMARC Group reports, the Saudi market of weight loss products touched the level of USD 1.28 billion in 2020. It is predicted to increase at a rate of 6.3% during the next five years [22].

This study is not without limitations. First, the sampling method was convenience, and therefore, it may not be appropriate to generalize the findings of this study. Secondly, no female pharmacist was enrolled in the study. Although an explanation is provided for this occurrence, having female pharmacists enrolled even in lesser numbers, such as the work of Alshahrani, would have provided the means for gender-based analysis. Lastly, the sampling carried out in the study rendered >80% of samples from pharmacies located in two cities, while few were from the rest. This may have negated the effect of the pharmacy location on the dispensing/selling of weight loss products. Therefore, further studies are recommended to evaluate the impact of location and gender of pharmacists on the dispensing/selling of weight loss products.

## 5. Conclusions

Based on the findings of this study, several varieties of tea followed by orlistat were the most used products for weight loss. Community pharmacists appeared most knowledgeable about the effectiveness and adverse effect of orlistat. The pharmacists seemed to be aware of the effectiveness of other weight loss products; however, their knowledge about the potential adverse effects of those products was unsatisfactory. These findings encore previous reports noting that the knowledge of community pharmacists, regarding commonly used weight loss products, is inadequate. Further education in this domain is required among community pharmacists.

## Figures and Tables

**Table 1:** Background characteristics.

Variables	Frequency	Percent
**Time (in minutes)**		
Up to 5	40	40.0
More than 5	60	60.0
**Experience**		
Up to 5	48	48.0
More than 5	52	52.0
**Gender of buyers**		
Females	80	80.0
Males	5	5.0
Both	15	15.0
**Buyers’ age**		
18–40	80	80.0
Above 40	20	20.0
**Location of the pharmacy**		
Khobar	31	31.0
Al-Ahsa	58	58.0
Dammam	8	8.0
Dhahran	3	3.0
**Asked about patient’s medical history before dispensing/selling a product**		
Always	65	65.0
Sometimes	30	30.0
Never	5	5.0

**Table 2:** Average packs sold for weight loss products per month.

Products	Mean (SD)	Median (IQR)	Range
Al-Diafa Tea	13.02 (13.55)	10 (13.75)	0 to 80
Green tea	10.86 (12.99)	6 (11.5)	0 to 80
Jamue tea	10.82 (14.32)	6 (9)	0 to 80
Orlistat	5.47 (4.98)	4 (5)	0 to 30
AppleFit (Apple cider vinegar)	4.67 (6.27)	3 (4)	0 to 35
Metformin	4.25 (8.22)	1 (5)	0 to 60
Chromium	2.06 (5.57)	0.5 (2)	0 to 44
FormoLine	1.49 (2.54)	1 (2)	0 to 20
Diet power	1.38 (2.31)	0 (2)	0 to 10
Opuntia Capsules	1.37 (4.54)	0 (1.75)	0 to 44
Super citrimax	1.06 (3.69)	0 (0)	0 to 30
Ripped power	0.75 (2.34)	0 (0.38)	0 to 20

Legend: FormoLine contains polymer Of D-Glucosamine, Al Diafa slimming tea contains *Cassia angustifolia* (Senna), Jamue tea contains *Tea folium* 80%, *Parameriae extractum* 6%, *Foeniculi extractum* 4%, *Guazumae extractum* 6%, *Curcumae extractum* 4%), Super citrimax contains hydroxycitrate complex extracted from *Garcinia Cambogia*, Ripped power contains L-carnitine L-tartrate, CLA (conjugated linoleic acid), robusta coffee extract (as *C. canephora robusta*) (fruit), rose hip extract (as *Rosa canina*), kelp (as *Laminaria digitata*) (stem and leaf).

**Table 3:** Effectiveness of weight loss products according to pharmacists.

Effectiveness	Yes	Sometimes	Often	No	Not Available
**FormoLine**	16 (16%)	17 (17%)	15 (15%)	3 (3%)	49 (49%)
**Orlistat**	71 (71%)	11 (11%)	14 (14%)	1 (1%)	3 (3%)
**Chromium**	11 (11%)	28 (28%)	8 (8%)	16 (16%)	37 (37%)
**Opuntia Capsules**	13 (13%)	27 (27%)	5 (5%)	16 (16%)	39 (29%)
**Diet power**	21 (21%)	18 (18%)	7 (7%)	3 (16%)	51 (51%)
**AppleFit (Apple cider vinegar)**	50 (50%)	22 (22%)	12 (12%)	0 (0%)	16 (16%)
**Metformin**	37 (37%)	19 (19%)	9 (9%)	6 (6%)	29 (29%)
**Green Tea**	25 (25%)	43 (43%)	7 (7%)	12 (12%)	13 (13%)
**Al-Diafa Tea**	28 (28%)	41 (41%)	14 (14%)	4 (4%)	13 (13%)
**Jamue Tea**	35 (35%)	28 (28%)	13 (13%)	4 (4%)	20 (20%)
**Super Citrimax**	11 (35%)	10 (10%)	2 (2%)	0 (0%)	77 (77%)
**Ripped power**	15 (15%)	9 (9%)	2 (2%)	0 (0%)	74 (74%)
**Sugar substitutes**	45 (45%)	33 (33%)	11 (11%)	11 (11%)	0 (0%)

Legend: FormoLine contains polymer Of D-Glucosamine, Al Diafa slimming tea contains *Cassia angustifolia* (Senna), Jamue tea contains *Tea folium* 80%, *Parameriae extractum* 6%, *Foeniculi extractum* 4%, *Guazumae extractum* 6%, *Curcumae extractum* 4%), Super citrimax contains hydroxycitrate complex extracted from *Garcinia Cambogia*, Ripped power contains L-carnitine L-tartrate, CLA (conjugated linoleic acid), robusta coffee extract (as *C. canephora robusta*) (fruit), rose hip extract (as *Rosa canina*), kelp (as *Laminaria digitata*) (stem and leaf).

**Table 4:** Responses of pharmacists regarding adverse effects of weight loss products.

Products	Responses Regarding Anticipated Adverse Effects	Frequency	Percent
FormoLine	No knowledge	91	91.0
	NVD, bloating, abdominal pain and constipation	6	6.0
	Others (liver disease, hyperthermia, insomnia)	3	3.0
Orlistat	No knowledge	12	12.0
	Fatty/oily spots	14	14.0
	NVD, bloating, abdominal pain and constipation	43	43.0
	Steatorrhea	21	21.0
	Hypertension	1	1.0
	Liver disease	6	6.0
	Kidney disease	1	1.0
	More than one side effect	2	2.0
Chromium	No knowledge	93	93.0
	NVD, bloating, abdominal pain and constipation	2	2.0
	Hypertension	3	3.0
	Pancreatitis	2	2.0
Opuntia Capsules	No knowledge	97	97.0
	NVD, bloating, abdominal pain and constipation	3	3.0
Diet power	No knowledge	93	93.0
	NVD, bloating, abdominal pain and constipation	1	1.0
	Hypertension	1	1.0
	Others (drowsiness, insomnia)	3	3.0
	More than one side effect	2	2.0
AppleFit (Apple cider vinegar)	No knowledge	92	92.0
	NVD, bloating, abdominal pain and constipation	6	6.0
	Others	1	1.0
	Kidney problems	1	1.0
Metformin	No knowledge	59	59.0
	NVD, bloating, abdominal pain and constipation	21	21.0
	Hypoglycemia	16	16.0
	Obesity	3	3.0
	More than one side effect	1	1.0
Green Tea	No knowledge	65	65.0
	NVD, bloating, abdominal pain and constipation	31	31.0
	Others (increased urination, spasm, electrolyte imbalance)	4	4.0
Al-Diafa Tea	No knowledge	50	50.0
	NVD, bloating, abdominal pain and constipation	48	48.0
	Others (spasms, electrolyte imbalance)	2	2.0
Jamue Tea	No knowledge	72	72.0
	NVD, bloating, abdominal pain and constipation	26	26.0
	Steatorrhea	1	1.0
	Others (electrolyte imbalance)	1	1.0
Super Citrimax	No knowledge	100	100.0
Ripped power	No knowledge	96	96.0
	NVD, bloating, abdominal pain and constipation	2	2.0
	Insomnia	2	2.0

Legend: FormoLine contains polymer Of D-Glucosamine, Al Diafa slimming tea contains *Cassia angustifolia* (Senna), Jamue tea contains *Tea folium* 80%, *Parameriae extractum* 6%, *Foeniculi extractum* 4%, *Guazumae extractum* 6%, *Curcumae extractum* 4%), Super citrimax contains hydroxycitrate complex extracted from *Garcinia Cambogia*, Ripped power contains L-carnitine L-tartrate, CLA (conjugated linoleic acid), robusta coffee extract (as *C. canephora robusta*) (fruit), rose hip extract (as *Rosa canina*), kelp (as *Laminaria digitata*) (stem and leaf).

## Data Availability

Not applicable.
